# Longitudinal natural history of type I spinal muscular atrophy: a critical review

**DOI:** 10.1186/s13023-020-01356-1

**Published:** 2020-04-05

**Authors:** Eugenio Mercuri, Simona Lucibello, Marco Perulli, Giorgia Coratti, Roberto de Sanctis, Maria Carmela Pera, Marika Pane, Jacqueline Montes, Darryl C. de Vivo, Basil T. Darras, Stephen J. Kolb, Richard S. Finkel

**Affiliations:** 1 Paediatric Neurology, Catholic University, Rome, Italy; 2 Centro Clinico Nemo, Policlinico Gemelli, Fondazione Policlinico Universitario Agostino Gemelli IRCCS, Rome, Italy; 3 Departments of Rehabilitation and Regenerative Medicine, Columbia University Medical Center, New York, USA; 4 Department of Neurology, Columbia University Medical Center, New York, USA; 5 Department of Neurology, Boston Children’s Hospital, Harvard Medical School, Boston, MA USA; 6 Department of Neurology, The Ohio State University Wexner Medical Center, Columbus, OH USA; 7 Department of Biological Chemistry & Pharmacology, The Ohio State University Wexner Medical Center, Columbus, OH USA; 8 Nemours Children’s Hospital, University of Central Florida College of Medicine, Orlando, USA

**Keywords:** Spinal muscular atrophy, Natural history, CHOP INTEND

## Abstract

**Background:**

The advent of new therapies in spinal muscular atrophy (SMA) has highlighted the need to have natural history data for comparison. Natural history studies using structured assessments in type I however are very limited. We identified and reviewed all the existing longitudinal history data in infants with type I SMA first assessed before the age of 7 months with the Children’s Hospital of Philadelphia Infant Test of Neuromuscular Disorders (CHOP INTEND).

**Main text:**

Three longitudinal natural history studies, two performed in the United States and one in Italy, were identified. The different study design of these three studies made it possible for the cumulative dataset to include the full spectrum of severity; from infants with neonatal onset to those with a milder phenotype that were not always included in the individual natural history studies. The cumulative analysis confirmed that, even in a larger cohort, there was never an improvement on the CHOP INTEND over time. This was true for all the infants, irrespective of their age or baseline CHOP INTEND scores. Infants with neonatal onset had low CHOP INTEND scores and a fast decline. The relatively large number of patients allowed us to calculate the rate of progression in subgroups identified according to *SMN2* copy number and baseline CHOP INTEND scores.

**Conclusion:**

A detailed understanding of the existing data is important, as it will be difficult to acquire new systematic longitudinal history data because of the availability of disease modifying therapies. The cumulative findings in this review help to better understand the variability of natural history data in untreated patients and will be of use for comparison to the real world patients treated with the recently approved therapies that have shown encouraging results in clinical trials.

## Background

Until recently most studies on spinal muscular atrophy (SMA) focused on the impact of palliative versus more proactive approaches on survival [1–3]. With improved standards of care [4, 5] and the recent clinical trials, there has been an increasing interest in identifying functional measures that could be used in weak infants, such as the Children’s Hospital of Philadelphia Infant Test of Neuromuscular Disorders (CHOP INTEND) [6, 7]. Only few studies have reported longitudinal changes using the CHOP INTEND [4, 8, 9] confirming that decline in motor function is invariably observed in type I infants. Each of these studies has a limited number of patients and it has become important to assess all the available data collected prior to the availability of therapies. This would help to better understand the variability in the natural history (NH) data and to make a comparison with the clinical trials and the ‘real world’ data that are steadily becoming available following the approval of Nusinersen, and, recently, of Zolgensma in the USA.

The aim of this study was to review and merge all the available data reporting longitudinal changes in the CHOP INTEND scores in type I SMA infants, focusing on those first assessed before the age of 7 months. As this scale has also been used in a large clinical trial with a sham group [10], which also included infants younger than 7 months of age, we also aimed at establishing how the sham data compared to the ones obtained as part of natural history studies.

## Main text

### Search approach

A comprehensive search of the following electronic databases was performed: MEDLINE, CINAHL, PsycINFO, and EMBASE.

The primary search terms ‘spinal muscular atrophy’ was combined with keywords ‘infants’, ‘type I’, ‘natural history’, ‘CHOP INTEND’. All electronic searches were limited to the English language and to publication years 2010 to May 2019. Reference lists of relevant articles were searched to identify any other further studies including the selected topics.

Furthermore, we also searched for the current or previous clinical trials in type I SMA, looking for placebo/sham arms using the CHOP INTEND.

To be included, studies had to meet the following inclusion criteria: (1) to include longitudinal data using the CHOP INTEND scale; (2) to include infants assessed below the age of 8 months; (3) to have a genetically proven diagnosis of SMA.

The titles and abstracts of articles were screened by the first authors.

Individual data were extracted and when needed, additional information were requested to the authors of the selected papers.

### Data extraction

Three longitudinal natural history studies using the CHOP INTEND were identified. Two were performed in the United States by the PNCR network [4] and the NeuroNEXT network [8, 11], and the other in Italy from a single center [9].

The three studies had different designs, but all included type I infants with genetically confirmed diagnosis of SMA assessed with the CHOP INTEND.

The PNCR study was a mixed retrospective and prospective study including 34 patients with type I of age ranging between 5 and 59 months [4]. Details of the SMN2 copies were only partially available and additional details on SMN2 copies were added as since the publication of the study they had become available. The NeuroNEXT study was a prospective study including 26 infants, all enrolled below the age of 7 months assessed with multiple scales [8].

The Italian single centre study was also partly retrospective and partly prospective including 37 infants with two copies all first seen below the age of 8 months and 3 patients seen between 8 and 12 months with 3 copies [9]. Details of the SMN2 copies were only partially available and additional details on SMN2 copies were added as since the publication of the study they had become available.

The following information was collated: patient baseline characteristics and demographics including, when available, Survival of Motor Neuron 2 (*SMN2)* copy number; change from baseline in CHOP INTEND. All data for individual patients from the three natural history studies were available in the papers or were made available on request.

We also identified a clinical trial that used the CHOP INTEND in type I SMA infants enrolled below the age of 7 months with a placebo (sham) arm of 41 patients [10].

**Fig. 1:**
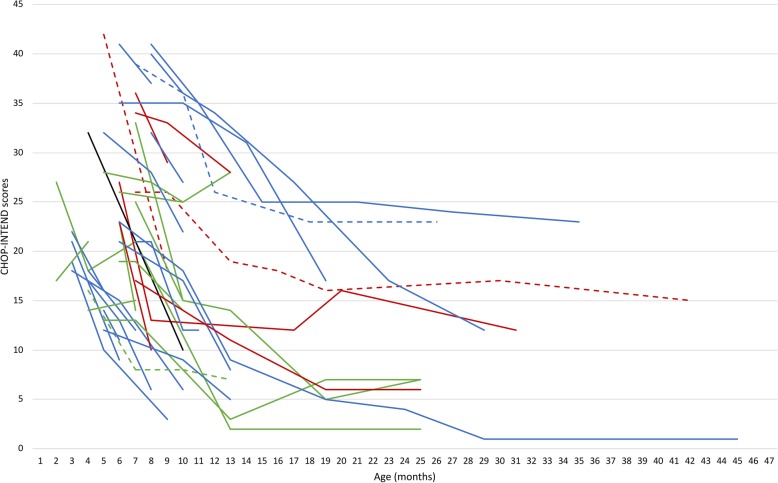
Individual CHOP-INTEND details of the 38 infants included: Figure shows the individual details of the 38 infants included: in green NeuroNEXT (Kolb et al., 2018); in red PNCR (Finkel et al., 2014), in blue Italian group (De Sanctis et al., 2018). Dotted line represent 3 SMN2 copies, black line 4 SMN2 copies

**Fig. 2:**
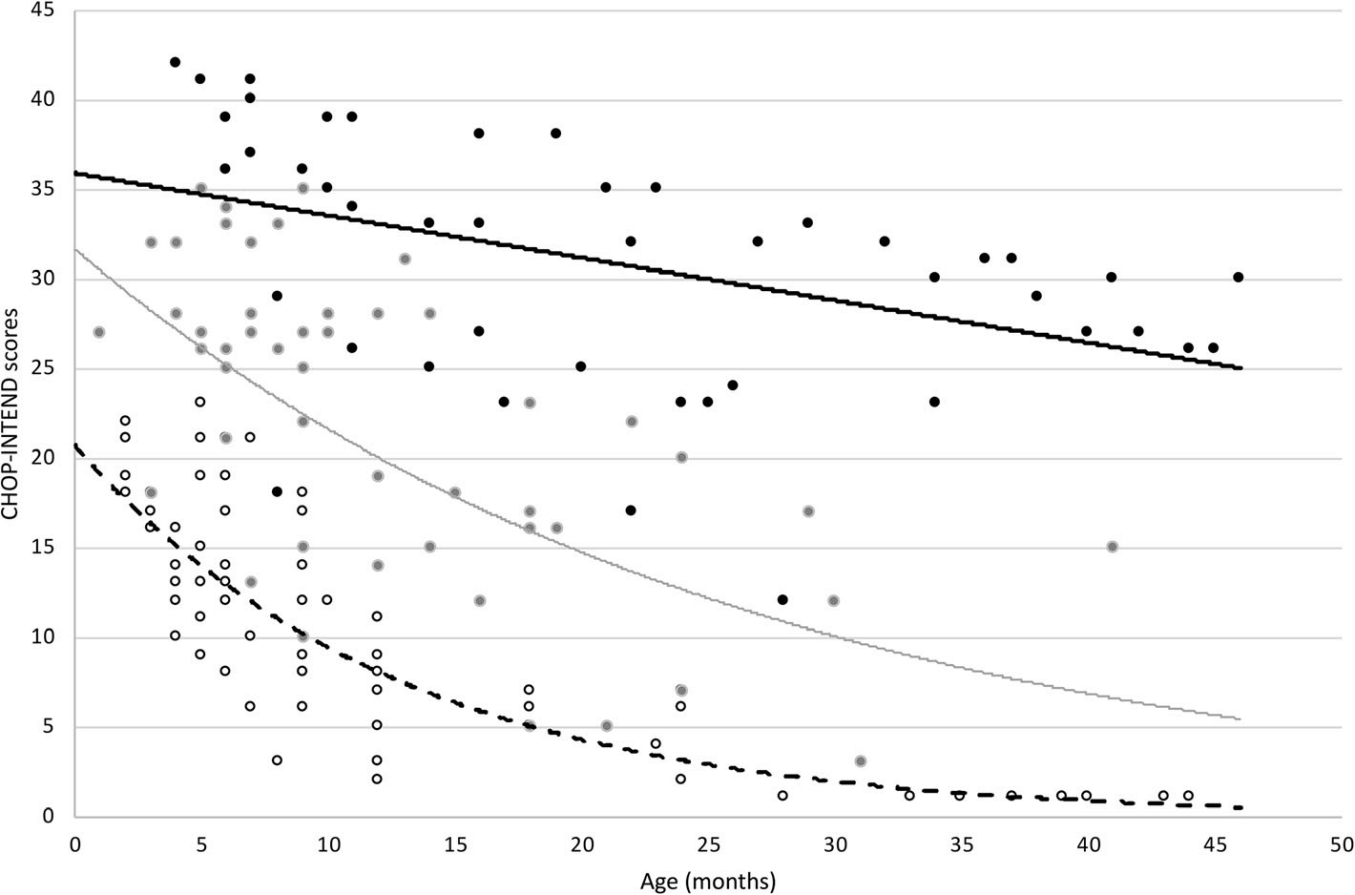
Details of NH patients selected by ENDEAR criteria: Figure shows CHOP-INTEND scores by age of NH patients with 2 SMN2 copies with onset after the neonatal period. [•] represent patient with CHOP-INTEND baseline scores above 25; [•] represent those with baseline score between 25 and 35; [•] represent those with baseline score below 35. The interpolation line represents the CHOP-INTEND progression subdivided by baseline score (—) if above 35; (—) if between 25 and 35; (--) if below 25

Individual details of all the patients are fully shown in Figs. 1, 2 and Table 1.

### Statistical analyses

Simple descriptive analysis was performed. Data were analyzed looking at changes in all the patients reported in the different natural history studies who were first assessed before the age of 7 months and in subgroups of patients with different *SMN2* copy numbers.

A separate analysis was performed in a selected subgroup using similar inclusion criteria to those used in clinical trials, i.e. including all the patients with two *SMN2* copies, but excluding the ones with early neonatal onset (up to 28 days from birth). Multiple polynomial regression was performed to compare the clinical trend in patients divided into three groups according scores at baseline (below 25, between 25 and 35, above 35) and including the age at baseline as predicting values.

### Natural history studies

The PNCR study included 34 type I SMA, of whom 14 classified as ‘recent’, assessed within 3 months from diagnosis and 20 as ‘chronic’, examined after 3 months from diagnosis [4]. Infants with the most severe neonatal form (1.1 or 1A) were not included. Seventeen subjects were evaluated on at least 2 occasions using the CHOP INTEND. In the original dataset 8 of the 17 were first assessed below the age of 7 months. These 8 were included in our analysis. Three infants had 2 *SMN2* copies of, 3 had 3 copies, 1 had 4 *SMN2* copies and in 1 SMN2 copy number was not available.

The NeuroNEXT study was a prospective study including 26 infants with SMA who were enrolled at 6 months of age or younger and followed for the first 2 years of life [8]. Because of the design of the study, that used the TIMPSI as a screening tool for the choice of assessment, only infants with TIMPSI scores less than 41 were assessed with the CHOP INTEND. This resulted in 12 of the 26 assessed using the CHOP INTEND who also had at least two consecutive assessments. Ten of the 12 had 2 *SMN2* copies and in the remaining two *SMN2* copy number was not available. The Italian single centre study was a partly retrospective and partly prospective study including 22 infants [9]. Eighteen of the 22 were assessed below the age of 7 months using the CHOP INTEND. Eight of the 18 infants had the severe form with neonatal onset. All infants had two copies of *SMN2*, one had three copies.

### Clinical trials

Only one published clinical trial using the CHOP INTEND as part of a randomized placebo controlled study was available. The placebo (sham) arm of the ENDEAR study included 41 infants with two *SMN2* copies assessed between the age of 30 and 262 days [10]. No individual data were available but details of the baseline and of the patients improving one or more points were reported in the paper and in the supplemental material.

### Cumulative natural history data

The cumulative ‘natural history’ cohort includes 38 infants with type I SMA infants examined before the age of 7 months, 30 with 2 copies, 4 with 3 copies, 1 with 4 copies. *SMN2* copy number was not determined in 3 infants.

Figure 1 shows the individual details of the 38 infants included.

### Patients with 2 SMN2 copies

The 30 patients with two copies were subdivided according to their baseline CHOP INTEND score:

Seventeen had a score below 25 and included all the severe early onset reported in the Italian group and 8 others with similar low scores but first assessed after the first months.

Nine had scores between 25 and 35 and only 4 infants had scores above 35. Only 2 of these 30 patients had a baseline CHOP INTEND score above 40 (both were 42).

### Patients with 2 SMN2 copies with neonatal onset

The 8 patients with neonatal onset had, with one exception, all scores below 25 and a fast decline. The rate of progression was a decline of a mean of 1.71 points/month.

### Patients with 2 SMN2 copies with onset after the neonatal period

The 22 infants with onset after the neonatal period had a more variable progression (Fig. 2).

The group with the lowest scores (below 25) (*n* = 8) all showed a fast decline and only two survived beyond 13 months. The rate of progression in the first year after baseline was a decline 1.02 points/month.

The group with scores between 25 and 35 (*n* = 10), also showed a rapid decline with a more variable duration of follow up. The rate of progression in the first year after baseline was a mean decline of 1.28 points/month.

Only 4 infants with 2 SMN2 copies had scores above 35. The rate of progression in the first year after baseline was a mean decline of 1.32 points/month. Age at baseline predicts rate of decline (*p* = 0,020). The difference between the subgroups subdivided according to baseline scores was significant (*p* < 0.001).

### Patients with 3 SMN2 copies

The 4 patients with three copies are equally distributed in the three groups according to baseline score: one falls into the group with baseline score between 25 and 35, two had a CHOP score at baseline above 35 (one above 40), one below 25. The rate of progression in the first year after baseline was a mean decline of 0,03 points/month.

### Patient with 4 SMN2 copies

The unique patient with 4 copies scored 32 at baseline showing a rate of progression of 3.66 points/month.

### ENDEAR sham group

The sham group included 41 infants, all with 2 SMN2 copies, and with onset after neonatal period. Table 1 shows details of their demographics. They were all screened between 1 month and 7 months with a first CHOP INTEND performed within 6 weeks. Their CHOP Intend baseline scores were 28.43 ± 7.56 (mean ± SD). Only 2 had scores above 40.

### Natural history versus sham group

Table 1 and Fig. 2 show details of the demographics of the sham group and of the subgroup of NH infants selected according to the same inclusion criteria used in the comparison clinical trial. A t-test of the mean CHOP INTEND scores at baseline showed no difference between the two cohorts T = 0.67 *p* > 0.05. An increase of at least 1 point was found in none of the 22 NH patients and in one of the 37 in the control group of the ENDEAR study (3%).

**Table 1:** demographics details of the sham group and of NH infants subgroup

	NH DATA	PLACEBO
**Number of patients***(n)*	22	41
**Age at baseline**		
*Mean (days)*	154	181
*Range (days)*	30–210	30–262
**CHOP INTEND scores***(mean + SD)*	27.36 + 8,54	28.42 + 7.56
**CHOP INTEND < 20***n/tot (%)*	4/22 (18%)	4/41 (10%)
**CHOP INTEND 20–40***n/tot (%)*	16/22 (73%)	34/41 (83%)
**CHOP INTEND > 40***n/tot (%)*	2/22 (9%)	3/41 (7%)

## Conclusions

Only few studies have reported longitudinal natural history data in type I SMA using structured functional assessments. Only one study used the HINE-2 (Hammersmith infant neurological examination – section 2) showing a decline in the developmental aspects explored by the scale. Another study showed the TIMPSI but in a restricted number of patients, also showing a progressive decline.

Most of the studies have used the CHOP INTEND scale [4, 8, 9]. These studies described for the first time the range of changes, suggesting some variability in the results that were possibly related to a number of factors, including baseline values, age, duration of symptoms, and the severity of the phenotype with associated respiratory and feeding comorbidities. The first study reporting longitudinal assessments highlighted how the changes differed according to the age at which the infants were assessed [4]. Those assessed within a few months after diagnosis generally have relatively higher scores and a faster decline than those assessed after a few months, when less functional abilities are generally present and there is a slower decline as there are fewer points to lose on the scale. The faster decline in younger infants has also been confirmed by subsequent studies [8, 9]. While each of the previous studies has strongly contributed to our understanding of the progression of the disease, they all had different study design and inclusion criteria and the possibility to compare data was limited because of differences in the cohorts studied.

In the present study, we included all the published studies and all the patients longitudinally assessed using the CHOP INTEND by the age of 7 months. The advantage of combining data form different papers with different inclusion/exclusion criteria allowed to overcome some possible bias related to the criteria used in individual studies, such as exlusion of patients with neonatal onset [4] or TIMPSI scores above 41 [8], allowing to have an overall final cohort with a larger spectrum of type I patients than those reported in individual studies.

Furthermore, the possibility to retrieve additional information from the original SMA datasets was useful in order to obtain more homogenous data and thus to conduct further analysis in a larger cohort and to better define possible trajectories. Additional individual details included age at baseline, that was not always available in the original papers, or other information, such as *SMN2* copy numbers that that were only partially available at the time of publication in the PNCR and in the Italian study. These details also allowed to better stratify the cohort according to copy number and to apply the same inclusion criteria used in the published clinical trial for a more appropriate comparison with the sham group. When the results of the three studies were combined using similar criteria, it was obvious that, even if collected in different countries, there were no obvious differences among the datasets, this probably reflecting that all the participating centers followed similar international standards of care [12, 13].

The different study design of the three studies made it possible for the cumulative dataset to include also infants with neonatal onset and those with a milder phenotype that were not always included in the individual studies. Our cumulative analysis confirmed that, even in a larger cohort, there was never a sustained improvement of the CHOP INTEND scores over time. This was true for all the infants, irrespective of their age or baseline CHOP INTEND scores. We were, however, able to observe variability in the progression of the disease, with infants with neonatal onset all having a low CHOP INTEND score and a more rapid and sharper decline than those with onset after the neonatal period.

In those with onset after the neonatal period and with 2 *SMN2* copies, the progression showed some variability with patients with higher baseline CHOP INTEND scores losing more points/ month than those with a lower baseline score. This probably reflects the fact that patients with higher scores had achieved more activities that were then progressively lost, i.e. they had more to lose. A baseline CHOP INTEND score greater than 40 points was found only in 2/22 NH infants and in 2/41 in the Sham cohort. This observation supports the recent opinion that an improvement up to and above 40 points is very uncommon in SMA type I infants with 2 copies of SMN2 and would indicate a favorable treatment effect beyond what has been observed in untreated patients.

Only four infants had 3 copies and appeared to have a less rapid decline but these results are limited as the majority of the patients who had 3 copies in the original papers were often first assessed after 7 months and were therefore not included in this study. These observations support the current thinking that clinical trials for SMA type I should be limited to participant with 2 copies of *SMN2* or to include those with 3 copies in a separate cohort.

Although individual longitudinal changes were not available in the original publication reporting the results of the ENDEAR clinical trial, we were able to demonstrate that, when using similar inclusion criteria, the baseline CHOP INTEND scores were similar. Furthermore, the comparison also suggests that in type I infants there is no obvious evidence of a sustained placebo effect over the duration of the trial as the number of responders in the placebo arm and in the NH group was similar. In the placebo group of the ENDEAR study an increase of at least 1 point was observed in only one patient (3%) and in none of the NH patients with similar inclusion criteria. Even when we examined the whole cohort of NH infants, an increase of more than one point was only found in one of the 38 NH infants. This finding is apparently in contrast with previous observations in other diseases suggesting that placebo groups may show a different disease progression compared to NH both for a possible “placebo effect” and for a likely difference in standard of care when entering a clinical trial. The lack of obvious placebo effect is probably partly justified by the fact that in all three NH studies the patients were followed in tertiary care centers with full adherence to the updated standards of care recommendations and data were collected by trained observers who followed a trial-like protocol including reliability training sessions [14]. These findings strengthen what has been recently discussed in international forums - including scientists, advocacy groups and regulators - that in a severely progressive disease such as type I SMA, there is no need for placebo arms in future clinical trials [15].

Unfortunately, as only the PNCR study also included infants first assessed after 7 months, additional data in this age group would help to better understand the changes observed in treated patients outside the age range used in clinical trials that so far have shown promising results [16–18]. Our cumulative results confirm that, even when a larger cohort of type I infants is included, there is never a consistent functional improvement in type I SMA infants that, in contrast, is frequently observed in treated patients in different published or ongoing clinical studies [10, 19, 20] or in recent real world data [17, 18] following the administration of commercially available drugs. The CHOP INTEND baseline scores can help to predict of the trajectories of progression. Patients with scores less than 25 showed the most rapid decline, those with scores above 35 the slowest and those between 25 and 35 an intermediate progression. The difference between the 3 subgroups was significant (*p* < .001).

While we acknowledge that the number of natural history patients reported in each study are relatively few, our cumulative dataset is the largest dataset available and will provide an important reference for the interpretation of new real-world data from treated patients that is proving to be challenging [21]. This appears to be particularly important as the possibility of collecting new systematic longitudinal natural history data in untreated patients is strongly reduced by the fact that currently most of the new diagnosed infants are immediately treated with the commercially available drugs or are enrolled in clinical trials.

## Data Availability

The datasets generated and/or analyzed during the current study are available from the corresponding author on reasonable request.

## Abbreviations

SMA: Spinal muscular atrophy
CHOP INTEND: Children’s Hospital of Philadelphia Infant Test of Neuromuscular Disorders
SMN2: Survival of motor neuron 2
PNCR: Pediatric neuromuscular clinical research
NH: Natural history

## References

[1] Bach JR (2007). Medical considerations of long-term survival of Werdnig-Hoffmann disease. Am J Phys Med Rehabil.

[2] Borkowska J, Rudnik-Schoneborn S, Hausmanowa-Petrusewicz I, Zerres K (2002). Early infantile form of spinal muscular atrophy (Werdnig-Hoffmann disease) with prolonged survival. Folia Neuropathol.

[3] Chung BH, Wong VC, Ip P (2004). Spinal muscular atrophy: survival pattern and functional status. Pediatrics.

[4] Finkel RS, McDermott MP, Kaufmann P (2014). Observational study of spinal muscular atrophy type I and implications for clinical trials. Neurology.

[5] Wang CH, Finkel RS, Bertini ES (2007). Consensus statement for standard of care in spinal muscular atrophy. J Child Neurol.

[6] Glanzman AM, Mazzone E, Main M (2010). The Children's Hospital of Philadelphia infant test of neuromuscular disorders (CHOP INTEND): test development and reliability. Neuromuscul Disord.

[7] Glanzman AM, O'Hagen JM, McDermott MP (2011). Validation of the expanded Hammersmith functional motor scale in spinal muscular atrophy type II and III. J Child Neurol.

[8] Kolb SJ, Coffey CS, Yankey JW, et al. Natural history of infantile-onset spinal muscular atrophy. Ann Neurol. 2017;82(6):883–91. 10.1002/ana.25101. Epub 2017 Dec 8. 10.1002/ana.25101 PMC5776712 29149772

[9] De Sanctis R, Pane M, Coratti G (2018). Clinical phenotypes and trajectories of disease progression in type I spinal muscular atrophy. Neuromuscul Disord.

[10] Finkel RS, Mercuri E, Darras BT (2017). Nusinersen versus sham control in infantile-onset spinal muscular atrophy. N Engl J Med.

[11] Kolb SJ, Coffey CS, Yankey JW (2016). Baseline results of the NeuroNEXT spinal muscular atrophy infant biomarker study. Ann Clin Transl Neurol.

[12] Finkel RS, Mercuri E, Meyer OH (2018). Diagnosis and management of spinal muscular atrophy: part 2: pulmonary and acute care; medications, supplements and immunizations; other organ systems; and ethics. Neuromuscul Disord.

[13] Mercuri E, Finkel RS, Muntoni F (2018). Diagnosis and management of spinal muscular atrophy: part 1: recommendations for diagnosis, rehabilitation, orthopedic and nutritional care. Neuromuscul Disord.

[14] Glanzman AM, Mazzone ES, Young SD (2018). Evaluator training and reliability for SMA global Nusinersen trials. J Neuromuscul Dis.

[15] Aartsma-Rus A, Balabanov P, Binetti L (2017). Stakeholder collaboration for spinal muscular atrophy therapy development. Lancet Neurol.

[16] Aragon-Gawinska K, Seferian AM, Daron A (2018). Nusinersen in patients older than 7 months with spinal muscular atrophy type I: a cohort study. Neurology.

[17] Pane M, Palermo C, Messina S (2018). Nusinersen in type I SMA infants, children and young adults: preliminary results on motor function. Neuromuscul Disord.

[18] Pechmann A, Langer T, Wider S, Kirschner J (2018). Single-center experience with intrathecal administration of Nusinersen in children with spinal muscular atrophy type I. Eur J Paediatr Neurol.

[19] Mendell JR, Al-Zaidy S, Shell R (2017). Single-dose gene-replacement therapy for spinal muscular atrophy. N Engl J Med.

[20] Chiriboga CA, Swoboda KJ, Darras BT (2016). Results from a phase 1 study of nusinersen (ISIS-SMN (Rx)) in children with spinal muscular atrophy. Neurology.

[21] Pechmann A, König K, Bernert G (2019). SMArtCARE - a platform to collect real-life outcome data of patients with spinal muscular atrophy. Orphanet J Rare Dis.

